# Debye–Waller coefficient of heavily deformed nanocrystalline iron^1^

**DOI:** 10.1107/S160057671700022X

**Published:** 2017-02-17

**Authors:** P. Scardi, L. Rebuffi, M. Abdellatief, A. Flor, A. Leonardi

**Affiliations:** a Department of Civil, Environmental and Mechanical Engineering, University of Trento, Trento, Italy; b Elettra-Sincrotrone Trieste S.C.p.A., Trieste, Italy; c Synchrotron Light for Experimental Science and Applications in the Middle East – SESAME, Allan, Jordan; d Department of Geological Sciences, Indiana University, Bloomington, Indiana, USA

**Keywords:** nanocrystalline materials, Debye–Waller coefficient, plastically deformed materials, temperature diffuse scattering, synchrotron radiation X-ray diffraction, EXAFS, molecular dynamics

## Abstract

Synchrotron radiation X-ray diffraction (XRD) patterns from an extensively ball-milled iron alloy powder were collected at 100, 200 and 300 K. The results were analysed together with those using extended X-ray absorption fine structure, measured on the same sample at liquid nitrogen temperature (77 K) and at room temperature (300 K), to assess the contribution of static disorder to the Debye–Waller coefficient (*B*_iso_). Both techniques give an increase of ∼20% with respect to bulk reference iron, a noticeably smaller difference than reported by most of the literature for similar systems. Besides good quality XRD patterns, proper consideration of the temperature diffuse scattering seems to be the key to accurate values of the Debye–Waller coefficient. Molecular dynamics simulations of nanocrystalline iron aggregates, mapped on the evidence provided by XRD in terms of domain size distribution, shed light on the origin of the observed *B*_iso_ increase. The main contribution to the static disorder is given by the grain boundary, while line and point defects have a much smaller effect.

## Abstract 2

Extensive deformation of an iron alloy powder increases the static disorder contribution to the thermal factor, with an increase of ∼20% in the Debye–Waller coefficient observed by both X-ray diffraction and extended X-ray absorption fine structure. Molecular dynamics simulations shed light on the underlying mechanisms, confirming the major role played by the grain boundary.

## 1. Introduction   

The Debye–Waller coefficient (*B*) provides a measure of the structural disorder in a material, directly related to the static and dynamic components of the atomic mean-square displace­ment (MSD, 〈*u* ^2^〉) (Krivoglaz, 1969 ▸; Kuhs, 2006 ▸). Like most properties, the MSD depends on the size of the crystalline domain (Clark *et al.*, 1965 ▸; Allen & De Wette, 1969 ▸). However, there are no simple and general rules to predict the MSD in nanocrystals. Besides domain size and shape, deviations from bulk MSD values depend on lattice defects and the environment surrounding the nanocrystals, *i.e.* whether they are embedded in a matrix or isolated, or are capped by contaminants or by suitable organic phases.

Free or weakly constrained metal nanoparticles often show a larger MSD, with a corresponding decrease in the average Debye temperature (Θ_D_) (Valiev *et al.*, 2000 ▸; Lu & Zhao, 1999 ▸). In simple monoatomic solids like cubic metal nanocrystals, the following relation holds between the MSD and Θ_D_ at sufficiently high temperatures (*T* > Θ_D_) to render quantum effects (zero-point vibrations) negligible (Willis & Pryor, 1975 ▸):
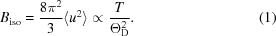
The MSD increase (and corresponding Θ_D_ decrease) is often attributed to a surface softening, as atoms on surfaces and at interfaces are under-coordinated. Low-energy electron diffraction (LEED) measurements on metals have shown that the surface Debye temperature is about 50% lower than the bulk value (Inagaki *et al.*, 1983 ▸; Clark *et al.*, 1965 ▸; Van Hove *et al.*, 1986 ▸), with a corresponding increase in *B*_iso_. This surface effect is responsible for the increase in the average MSD (decrease in the average Θ_D_), which in nanocrystals scales approximately as the surface/volume ratio, *i.e.* as 1/*D* (Inagaki *et al.*, 1983 ▸), where *D* is the diameter or any characteristic length of the crystalline domain. Mössbauer spectroscopy has shown a similar core/shell effect in both free iron nanocrystals (Von Eynatten & Bömmel, 1977 ▸) and ball-milled Cr–Fe alloy nanocrystalline powders, where the Debye temperature of the grain boundary region was found to be 100 K lower (*i.e.* about 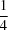 lower) than the bulk value (Kuwano *et al.*, 1992 ▸). Beyond the simplistic view of a sharp core(bulk)/shell(surface) model, molecular dynamics (MD) has shown quite clearly that the MSD is not constant across a metal nanoparticle. Rather than undergoing a sharp change from core to surface, the MSD increases steeply but continuously toward the surface layers, where the low coordination has an effect on the atomic displacement and vibrational properties (Gelisio *et al.*, 2013 ▸; Beyerlein *et al.*, 2012 ▸).

Besides the specific effect on the vibration dynamics of nanocrystals, the MSD increase is also related to a corresponding increase both in the thermal expansion coefficient (Yang *et al.*, 2006 ▸) and in the heat capacity at low temperature, where surface vibration modes are important (Michailov & Avramov, 2012 ▸; Bai *et al.*, 1996 ▸). The Debye–Waller (DW) coefficient of nanocrystals can therefore deviate considerably from the corresponding bulk perfect-crystal values and, in addition to the above-mentioned effects, different types of static disorder can be responsible for values in excess of the expected ones (Krivoglaz, 1969 ▸; Londsdale, 1968 ▸). More recently, coupled MD and X-ray powder diffraction (XRPD) simulations have suggested that static disorder in grain boundary regions contributes to both coherent and diffuse scattering (Leonardi *et al.*, 2013 ▸; Leonardi, Leoni, Li & Scardi, 2012 ▸), the latter resulting in a thermal-like step in the background of Warren plot profiles [Fig. 3 in the paper by Warren & Averbach (1950 ▸)]. Experimental measurements of the DW coefficient are therefore indispensable for assessing the presence and extent of the different contributing effects.

Despite the interest in and generality of the problem, the measured *B* values for nanocrystalline materials vary considerably in the literature, sometimes showing huge increments with respect to the reference values (Lu & Zhao, 1999 ▸). Values for ball-milled iron, for example, have been reported to be 110% (Lu & Zhao, 1999 ▸; Zhao, 2001 ▸) or even 300% (Azzaza *et al.*, 2015 ▸) higher than the reference DW coefficient for bulk iron (Butt *et al.*, 1988 ▸). The interpretation of such large increases in *B* is not always clear. Even if the role of surfaces and grain boundaries is well established (Van Hove *et al.*, 1986 ▸), large *B* values in ball-milled nanocrystalline materials have frequently been ascribed to some local strain (microstrain), a measure of which is obtained from diffraction line broadening (Azzaza *et al.*, 2015 ▸; Zhao, 2001 ▸; Sirdeshmukh *et al.*, 1993 ▸; Purushotham & Krishna, 2010 ▸), with no further justification or proof of a real cause–effect linkage.

The present paper investigates the DW coefficient of an extensively ball-milled iron alloy powder. The modelling of the synchrotron radiation XRPD patterns collected at three temperatures (100, 200 and 300 K), complemented by extended X-ray absorption fine structure (EXAFS) results for the same sample, provides values of the DW coefficient definitely smaller than those in the above-cited literature. With the support of simulations of nanocrystalline iron clusters made by MD, this work sheds light on the origin of the increased *B* with respect to bulk values and on the role of the correlated displacement of neighbouring atoms.

## 2. Experimental   

The studied sample is an Astaloy Mo powder (Fe–1.5 wt% Mo, FeMo), extensively ground (64 h) in a Fritsch P4 planetary ball mill. Details of the grinding process and the resulting powder can be found in the paper by Rebuffi *et al.* (2016 ▸) for the experimental part and electron microscopy, whereas the work of Broseghini *et al.* (2016 ▸) deals with a kinetic modelling of the mill used in the optimization of the grinding process.

XRPD data were collected on 11-BM, the powder diffraction beamline at the Advanced Photon Source (Argonne National Laboratory, Illinois, USA) based on the Debye–Scherrer geometry. The X-ray beam of nominal energy 30 keV (actual wavelength λ = 0.0413679 nm) was diffracted by an FeMo powder specimen loaded in a Kapton capillary (radius *R* = 0.15 mm), using a detector assembly consisting of 12 independent Si(111) analysers and as many scintillation counters. The 2θ sampling step was 0.005°, over a 2θ range from 0 to 55°, with a counting time of 0.3 s per step.

The capillary mount is particularly convenient for collecting reliable intensity values, provided that the absorption is sufficiently low so that data corrections can be avoided. In fact, expressions to correct the intensity for absorption are known (Maslen, 2006 ▸) but they require information on the density of the specimen, which is hardly known, especially in a spinning capillary. Therefore, to limit absorption the powder was diluted in carbon black and just lightly pushed into the capillary, enough to remain steady on spinning but still low density. Preliminary absorption measurements were made at 22.163 keV using X-rays from a sealed tube with an Ag anode (see the supporting information). On the basis of direct measurement of the linear absorption coefficient, μ, we could estimate μ*R* 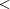 0.1 for the 30 keV of 11-BM, a value sufficiently low to make absorption corrections unnecessary. XRPD data on the same sealed capillary were collected at 100, 200 and 300 K, in sequence, using an air blower to condition the capillary temperature.

The instrumental profile (IP) was also evaluated experimentally, collecting the pattern of NIST SRM660a (LaB_6_; Cline *et al.*, 2000 ▸) under comparable conditions. The IP was parameterized in terms of the 2θ-dependent trends in width and shape of a pseudo-Voigt line profile fitting the experimental data (see the supporting information). The contribution from the Kapton capillary and air scattering was also carefully evaluated: the pattern of a blank capillary was fitted by seven pseudo-Voigt functions, enough to reproduce the pattern empirically, and the resulting model was adapted as background to the ball-milled FeMo data by means of a refinable scaling factor. Further details are reported in the supporting information.

The EXAFS measurements using the transmission mode were performed on the XAS beamline at the Elettra synchrotron in Trieste, Italy. The XAS beamline is installed on a bending magnet source and it is dedicated to X-ray absorption spectroscopy experiments between 2.4 and 27 keV (Di Cicco *et al.*, 2009 ▸). A homogenous pellet for the ground FeMo sample was prepared by mixing a fixed amount of FeMo together with poly(tetra­fluoro­ethylene) membrane and then the mixture was subjected to low applied pressure to prepare a solid disc. The EXAFS measurements were carried out at the *K* edge of Fe (*i.e.* *E* = 7112 eV) while the energy scan ranged from 6912 to 8638 eV, with an energy step varying from 0.2 eV at the near-edge region to 5 eV at the extremes of the spectrum. The incident and transmitted intensities were measured by two ionization chambers, before and after the sample, respectively. For better statistics, several scans were collected on the sample at room temperature and at liquid nitrogen temperature. Further details are reported in the supporting information.

## 3. Numerical simulations   

Atomistic simulations of a cluster of iron nanocrystals were performed *via* MD using the software *LAMMPS* (Plimpton, 1995 ▸) and the embedded atom method (EAM; Daw & Baskes, 1983 ▸; 1984 ▸). An ideally crystallographic microstructure of 50 iron crystallites was generated by dividing the space within a cubic box with periodic boundary conditions (PBCs) using a modified Voronoi tessellation algorithm (Leonardi, Scardi & Leoni, 2012 ▸) and filling each tessellation cell with a randomly oriented periodic structure [*i.e.* body-centred cubic (b.c.c.), 2.86650 Å unit-cell parameter]. The space tessellation was allowed to evolve (Leonardi *et al.*, 2013 ▸; Gross & Li, 2002 ▸; Xu & Li, 2009 ▸) by constraining the cluster to follow the log-normal distribution of diameters determined experimentally by XRPD (Rebuffi *et al.*, 2016 ▸). Thus, the ideal system was energy-minimized by iteratively adjusting atom coordinates, and then equilibrated with a 0.6 ns dynamics up to reaching a steady state at constant pressure (0 Pa) and temperature (300 K), using a Nose–Hoover style non-Hamiltonian thermo­stat (Martyna *et al.*, 1994 ▸; Parrinello & Rahman, 1981 ▸; Shinoda *et al.*, 2004 ▸; Tuckerman *et al.*, 2006 ▸) with a 1 fs time integration and Fe EAM pair potential (Mendelev *et al.*, 2003 ▸). Next, a set of uncorrelated arrangements of atomic positions in space (*i.e.* frames) were recorded at 2 ps time intervals for a 0.2 ns long dynamics (*i.e* a time trajectory of 100 frames). The time-average configuration was thus computed to cancel the dynamic component out of the lattice distortion field (Leonardi *et al.*, 2011 ▸; Leonardi, Leoni, Li & Scardi, 2012 ▸).

The PBCs employed in MD simulations were removed, recovering the continuity of the structure within the crystalline domains. Furthermore, the crystallites were rigidly translated by the PBC length so as to minimize the surface-to-volume ratio of the resulting polycrystalline microstructure (*i.e.* an average spherical shape). Next, XRPD patterns were simulated using the Debye scattering equation (DSE) (Debye, 1915 ▸; Warren, 1990 ▸; Gelisio *et al.*, 2010 ▸; Leonardi & Bish, 2016 ▸) for all the frame configurations and their average over the time trajectory. Finally, the profiles for the uncorrelated frames were summed to obtain an experimental-like XRPD pattern, accounting for an effective real-life measurement time. Normalized by the numbers of frames and atoms, respectively, the averaged positions (AP) and time-average (TA) profiles therefore differed only in the dynamic contribution to the line broadening (*i.e.* temperature diffuse scattering, TDS). XRPD patterns for each single crystal were also recorded while calculating the profiles for the whole microstructure. Thus, the XRD pattern for an ideal powder made of monodisperse domains like a randomly chosen crystallite was obtained, neglecting the contribution of inter-crystallite interference to the observed XRPD profiles (Leonardi *et al.*, 2013 ▸; Leonardi, 2015 ▸).

The radial pair distribution function (RPDF) was calculated over 100 frames along the MD trajectory, using the software *VMD* (Humphrey *et al.*, 1996 ▸; Levine *et al.*, 2011 ▸).

## 4. Results and discussion   

Diffraction data collected at the three temperatures (100, 200 and 300 K) were analysed by whole powder pattern modelling (WPPM), following a procedure outlined by Rebuffi *et al.* (2016 ▸). Line profiles of the b.c.c. FeMo phase [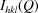] were modelled assuming their Fourier transform to be made up of three contributions, namely the IP [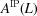], the finite domain size [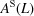], and the inhomogeneous strain or microstrain [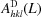] (Scardi, 2008 ▸):
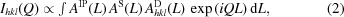
where *hkl* are the Miller indices of the modelled peak profile [*Q* = (4π/λ)sin(θ/2), θ being the scattering angle and λ the X-ray wavelength] and *L* is the Fourier length, the distance between any two scattering centres along [*hkl*]. The proportionality factor includes the usual trigonometric terms of the Lorentz polarization factor with known constants and the square modulus of the structure factor corrected for temperature effects by the DW factor (Warren, 1990 ▸). Crystalline domains were assumed to be spherical, with diameters distributed according to a log-normal distribution with the log-normal mean (μ) and standard deviation (σ) as refinable parameters.

The microstrain term can be expressed according to the Krivoglaz–Wilkens theory of dislocation line broadening (Krivoglaz, 1969 ▸; Wilkens, 1969 ▸, 1970 ▸), with the average dislocation density ρ and the effective outer cut-off *R*_e_ as refinable parameters. Dislocations are expected in the primary slip system of iron, {110}〈111〉 (Burgers vector modulus 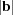 = (*a*_0_3^1/2^)/2 with the unit-cell parameter *a*_0_), for which the average contrast factor is known (D’Incau *et al.*, 2007 ▸) for edge- and screw-type dislocations, so that the fraction of edge-type dislocations, *f*_E_, can also be refined. However, as pointed out recently (Rebuffi *et al.*, 2016 ▸), this model tends to overestimate the dislocation density, with 1.5–2 dis­locations per domain, well beyond the evidence of transmission electron microscopy (TEM). In this specific sample, as is also probably true of many ball-milled metals, the inhomogeneous strain responsible for the anisotropic line broadening is only partly caused by line defects in the crystalline domains, whereas a large part of the effect is due to grain boundary regions, to which dislocations migrate in the deformation process, and to grain–grain interactions. It is therefore more appropriate to express the strain broadening result in terms of a Warren plot (Warren & Averbach, 1950 ▸), *i.e.* the r.m.s. displacement of pairs of unit cells, 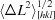, for any given distance *L* in the crystalline domain along the [*hkl*] scattering direction. This provides a quantitative measurement but leaves open the interpretation of the microstrain effect as resulting from one or more sources (Rebuffi *et al.*, 2016 ▸).

The main focus of this work is on the DW coefficient of the ball-milled FeMo powder, so the role of MSD/*B* was considered in detail. The DW coefficient appears in the traditional thermal factor depressing the Bragg intensity, which for a monoatomic cubic phase reads (Warren, 1990 ▸; Guinier, 1963 ▸)
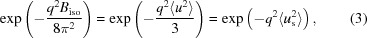
where 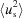 is the projection of 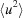 along the [*hkl*] scattering vector direction for the given diffraction peak. In addition to this term, *B* also appears in the diffuse scattering component, which can be included in WPPM following Warren’s theory (Warren, 1990 ▸), as modified by Beyerlein *et al.* (2012 ▸) to account for the phonon confinement effect enforced by the finite (small) domain size. Including TDS in the WPPM is important, as it gives more reliable *B* values as well as a more accurate modelling of the line profiles, without increasing the number of refined parameters.

Data modelling was done simultaneously on patterns collected at different temperatures, sharing the same microstructural (size–strain) model, while independently refining for each pattern the DW coefficient [*B*_iso_(*T*)] and the unit-cell parameter [*a*_0_(*T*)]. WPPM also included a second phase, a small fraction (∼2%) of austenitic face-centred cubic (f.c.c.) iron, undetected by previous studies. Besides a scaling factor, only the unit-cell parameter of the austenitic phase was added as a refinable parameter, all other microstructural and thermal parameters being shared with the main b.c.c. phase so as to limit the number of free parameters.

**Figure 1:**
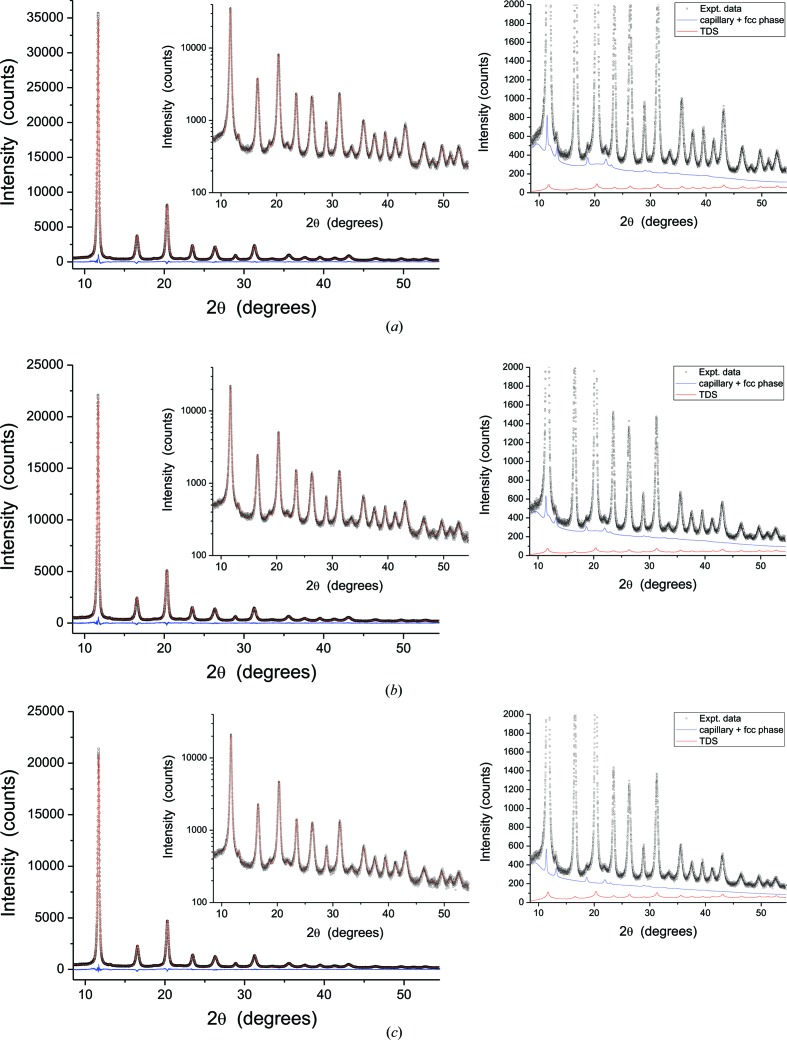
X-ray diffraction patterns of ball-milled FeMo powder at (*a*) 100 K, (*b*) 200 K and (*c*) 300 K. Experimental data (circles) are shown together with the modelling (red lines) and their difference (residual, blue lines below each plot). WPPM details are shown in the left-hand insets on a log scale, whereas the insets on the right show contributions from the capillary and f.c.c. minor fraction, and from the TDS.

The XRPD patterns of the ball-milled FeMo sample are displayed in Fig. 1 ▸ for the three studied temperatures [100, 200 and 300 K, respectively, in parts (*a*), (*b*) and (*c*)], together with the WPPM results. The insets on the left-hand side highlight details on a log scale, while the right-hand plots give a comparison of the experimental data with the TDS and the signal from the capillary, summed to the minor fraction of the f.c.c. phase. Further details of the TDS and capillary contributions are reported in the supporting information.

**Figure 2:**
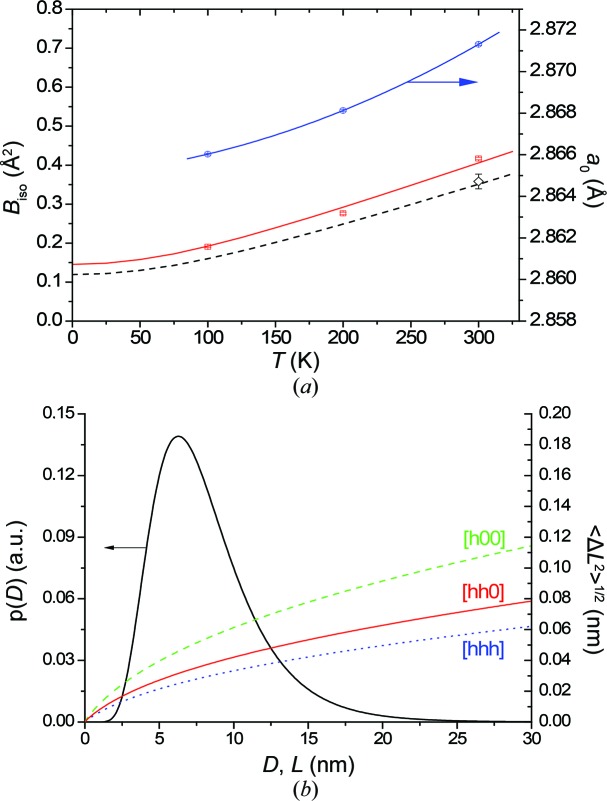
Temperature-dependent parameters from the modelling of Fig. 1 ▸. (*a*) *B*_iso_(*T*) (left-hand axis) and *a*_0_(*T*) (right-hand axis). The *B*_iso_(*T*) trend from the best fit to equation (4) (red line) is shown together with the literature data for bulk Fe (Butt *et al.*, 1988 ▸) (dashed line), whereas the *a*_0_(*T*) trend (blue line) is a parabolic function simply to guide the eye. (*b*) Domain diameter distribution (left-hand axis) and Warren plots (Warren, 1990 ▸) (right-hand axis); see text for details.

The results of WPPM are shown in Fig. 2 ▸(*a*) for the temperature-dependent parameters *B*_iso_(*T*) and *a*_0_(*T*), while the domain size distribution and Warren plot for three significant directions ([*h*00], [*hh*0] and [*hhh*]) are shown in Fig. 2 ▸(*b*). The mean domain size of 8.2 (2) nm is smaller than the value given by Rebuffi *et al.* (2016 ▸) of 9.3 (8) nm, as the present analysis includes the TDS, with the simultaneous modelling of patterns collected at three different temperatures with higher X-ray energy, and the austenitic phase. This minor fraction of the f.c.c. phase was probably stabilized by nickel contamination from the milling vial and could only be detected for the high-quality counting statistics of the 11-BM data. On the other hand, the r.m.s. displacement in the Warren plot is nearly identical to the results of Rebuffi *et al.* (2016 ▸).

The *B*_iso_(*T*) values for the ball-milled sample are about 20% higher than the reference values for bulk iron, which are shown in Fig. 2 ▸(*a*) as a dashed line, together with the value for pristine FeMo powder at ambient *T*: *B*_iso_(300 K) for pristine FeMo powder is 0.358 (10) Å^2^, whereas the literature value for bulk Fe is 0.35 (1) Å^2^ with Θ_D_ = 431 (6) K (Butt *et al.*, 1988 ▸), against 0.417 (3) Å^2^ for the ball-milled FeMo. It is worth mentioning here that the 1.5 wt% Mo in the ball-milled powder, equivalent to 0.88 at.% Mo, gives a negligible contribution to the average DW factor [using the rule of mixtures, *B*_iso_(300  K) = 0.349 Å^2^ for the bulk FeMo phase], so we refer to the pure iron data with no further specification in the context of this work.

Both trends in Fig. 2 ▸(*a*), *B*_iso_(*T*) and *a*_0_(*T*), show the expected nonlinearity towards the low-*T* limit, where quantum effects, and zero-point energy in particular, become relevant. Since the experimental evidence we could gather is limited to three data points, the only modelling of *B*_iso_(*T*) that we can credibly afford is with the Debye theory. In fact, MSD/*B* can be modelled as (Warren, 1990 ▸; Willis & Pryor, 1975 ▸)
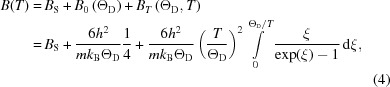
where *h* is the Planck constant, 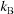 the Boltzmann constant and *m* the atomic mass, and with *B*_S_, the static component of the DW coefficient, and Θ_D_ as the parameters to be adjusted. The best fit, shown in Fig. 2 ▸(*a*) as a solid line, gives *B*_S_ = 0.02 and Θ_D_ = 420 K. It can be noted that the difference between the bulk reference (dashed line) and the ball-milled sample (solid line) tends to increase slightly towards higher (300 K) temperature, which is a detail already mentioned in the cited works based on LEED and Mössbauer spectroscopy. This is a consequence of the surface softening effect, which determines the lower Debye temperature with respect to the bulk reference.

**Figure 3:**
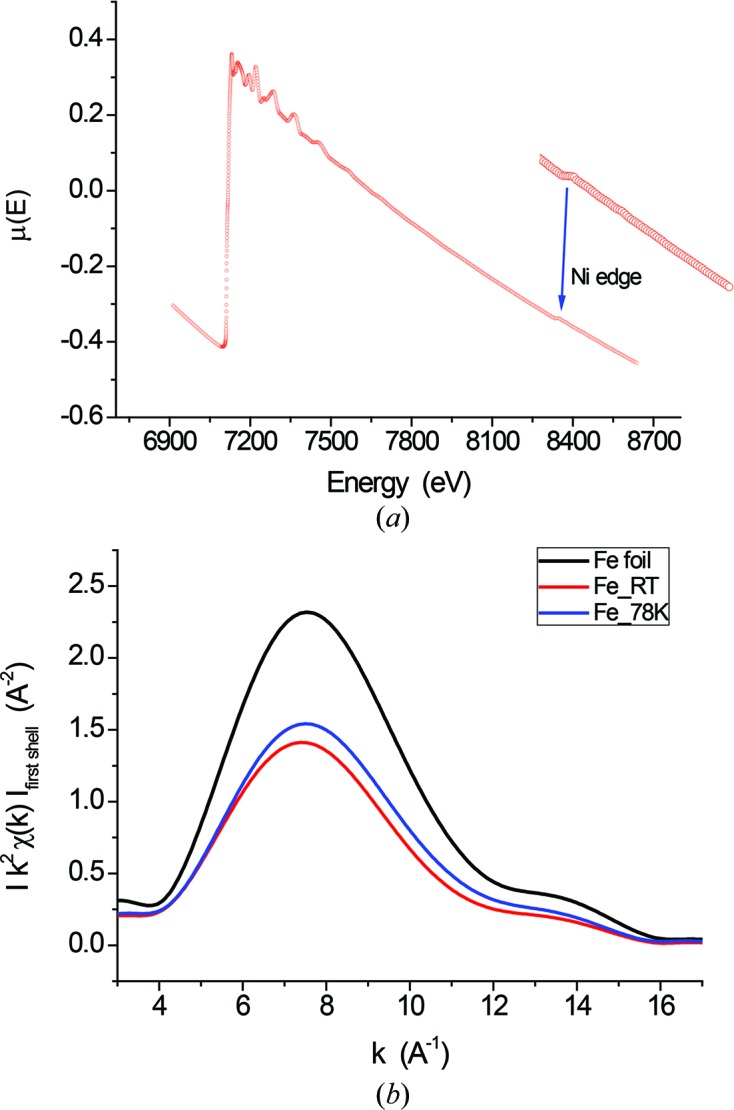
(*a*) EXAFS measurements around the Fe *K* edge at 7110 eV, with detail of the additional edge due to the nickel contaminant. (*b*) The amplitude signal of the back Fourier transform of *k* ^2^χ(*R*) corresponding to the first shell.

Similar evidence is provided by EXAFS measurements made on the same capillary with ball-milled FeMo powder and, for comparison, on a pure Fe thin foil used as standard. As shown in Fig. 3 ▸, the absorption was measured around the Fe *K* edge at 7.11 keV. The same measurement confirms the presence of Ni contamination from the mill (jar), which is deemed responsible for the formation of a minor fraction of austenitic γ phase.

EXAFS data were analysed considering the first coordination shell, using an Fe foil as standard material with known structural parameters (Sevillano *et al.*, 1979 ▸). According to the ratio method (Schnohr & Ridgway, 2015 ▸), the amplitude of the Fourier transform of the normalized EXAFS function χ(*k*) (Fig. 3 ▸ *b*) for the first shell of the ball-milled FeMo was divided by the corresponding amplitude for the foil; the result is a linear function of the square of the wavenumber vector modulus (*k*), the slope of which gives the difference in DW factors (see the supporting information for details).

The DW coefficients from EXAFS and XRPD have quite different meanings (Sevillano *et al.*, 1979 ▸; Fornasini, 2014 ▸; Dalba & Fornasini, 1997 ▸), the main difference arising from the different averages involved in the corresponding definitions. While the EXAFS value of our study refers to the first co­ordination shell (eight neighbours in ideal b.c.c. Fe), the XRPD value is an average over the whole system, which according to Debye’s model involves all 3*N* vibration modes in a b.c.c. domain with *N* iron atoms.

**Figure 4:**
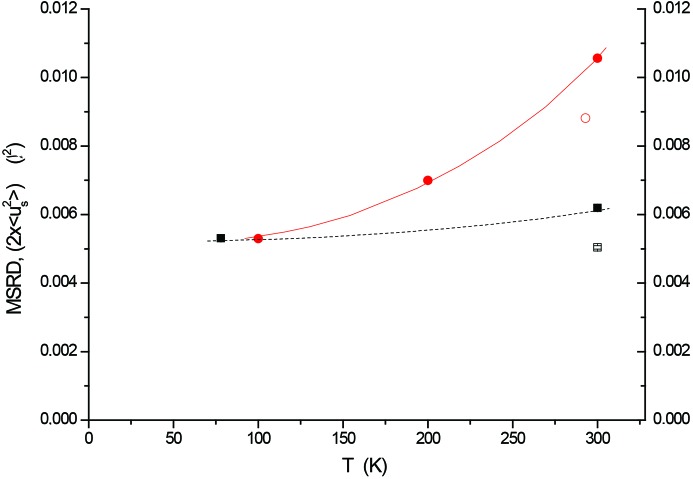
MSRD of the first Fe coordination shell from EXAFS measurements at 77 and 298 K (Figs. 2 ▸ and 3 ▸) (squares). The values of 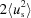 (circles) are obtained from the XRPD values of *B*_iso_ in Fig. 2 ▸(*a*) using equation (1). Filled symbols denote data for ball-milled FeMo, while open symbols denote data for the pure iron foil (EXAFS, square) and the literature value for bulk iron (circle) (Butt *et al.*, 1988 ▸).

The DW coefficient from EXAFS is the parallel mean-square relative displacement (MSRD), 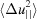, defined as the variance of the distribution of distances between absorbing and backscattering atoms (Calvin, 2013 ▸). For totally un­correlated atomic displacements (Einstein oscillators), the MSRD is equal to twice the MSD component projected along the direction joining absorbing and backscattering atoms, which is equivalent, to a good level of approximation, to 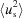, the DW coefficient provided by XRPD. In the general case where atomic displacements are correlated, the MSRD can be written as (Calvin, 2013 ▸; Fornasini, 2014 ▸)
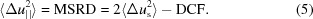
Here, the difference between twice the MSD and the MSRD defines the displacement correlation function (DCF). Experimentally determined 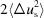, from *B*_iso_ values and equation (1), and the MSRD for the Fe first shell are shown in Fig. 4 ▸ as a function of temperature. While the first-shell MSRD is weakly affected by temperature, 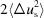 increases quite steeply, the difference between the two quantities being caused by the strong correlation of atomic displacements in the first coordination shell.

As shown by Jeong *et al.* (2003 ▸), the atomic vibrations of bulk iron can be described by a Born–von Karman (BvK) force model (Born & Huang, 1954 ▸). By solving the dynamic matrix using up to the fifth nearest-neighbour interatomic force parameters, the DCF of the first shell is 42% of 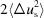. Then, according to equation (5), Butt’s value of 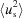 = 0.004408 Å^2^ for reference bulk iron (Butt *et al.*, 1988 ▸) corresponds to a first-shell MRSD = 2 × 0.004408 × (1 − 0.42) = 0.00511 Å^2^, in good agreement with the experimental EXAFS results of Sevillano and co-workers (0.00506 Å^2^; Sevillano *et al.*, 1979 ▸) and subsequent literature (Jiménez-Villacorta *et al.*, 2004 ▸).

If the same argument is used for ball-milled FeMo, a similar agreement is obtained between the first-shell MSRD values from the BvK model and EXAFS experiments. In more detail, the MSRD shows a 20% increment from the bulk reference [Fig. 4 ▸, open square, 0.00504 (5) Å^2^] to ball-milled FeMo [filled square at 300 K, 0.00619 (1) Å^2^], thus also confirming the effect of the static disorder component according to EXAFS, to the same extent shown by the XRPD results in Fig. 2 ▸(*a*).

The XRPD and EXAFS results for the DW coefficient are therefore in good agreement, pointing to a static disorder component compatible with LEED and Mössbauer observations (Inagaki *et al.*, 1983 ▸; Kuwano *et al.*, 1992 ▸). Quite differently, the literature data for *B*_iso_ appear overestimated (Zhao *et al.*, 2001 ▸; Azzaza *et al.*, 2015 ▸), probably because of experimental or modelling errors. XRD measurements of *B*_iso_ require a strict control of all factors – primarily absorption – affecting the diffracted intensity; moreover, as pointed out by Vetelino *et al.* (1972 ▸), accounting for the TDS in XRPD data modelling is important, and failing to do so could be a further reason for the quite high *B*_iso_ values reported in the literature (Lu & Zhao, 1999 ▸; Zhao *et al.*, 2001 ▸; Azzaza *et al.*, 2015 ▸; Sirdeshmukh *et al.*, 1993 ▸; Purushotham & Krishna, 2010 ▸).

**Figure 5:**
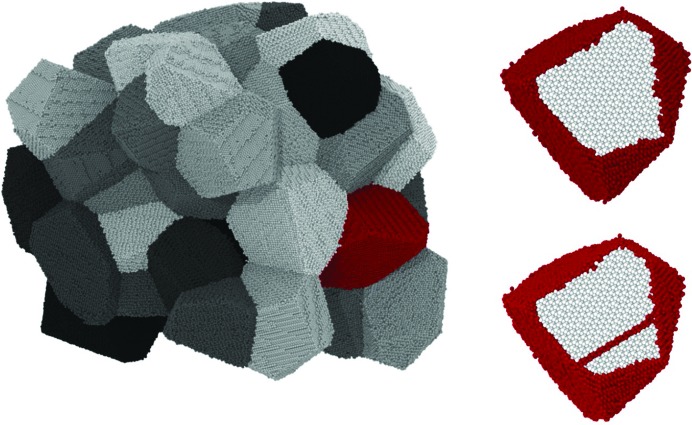
MD model of the studied system, made up of 50 grains of Fe with a size distribution mapped on the experimental result of WPPM shown in Fig. 2 ▸(*b*). On the right-hand side are shown detailed images of grain number 5, an average-size crystallite with 66 874 Fe atoms (top), and the same grain with an edge dislocation (bottom). See text for details.

To add a further point of view and interpretation of the experimental results, and to assess the origin of the observed increase in the DW coefficient, a system similar to the ball-milled FeMo was simulated by MD. As explained in §3, a cluster of 50 grains was generated by a modified Voronoi tessellation procedure (Leonardi *et al.*, 2013 ▸). Atomistic models of iron grains are polyhedra with the same volume as the spherical grains of the distribution of Fig. 2 ▸(*b*) refined by WPPM. Grain number 5, highlighted in red in Fig. 5 ▸, is an average-sized crystalline domain with 66 874 Fe atoms. The 50-grain cluster in Fig. 5 ▸ is shown with the detail of each grain, but in the actual MD simulations PBCs were enforced, so that each grain, and grain number 5 in particular, experienced an environment similar to that of the real material.

**Figure 6:**
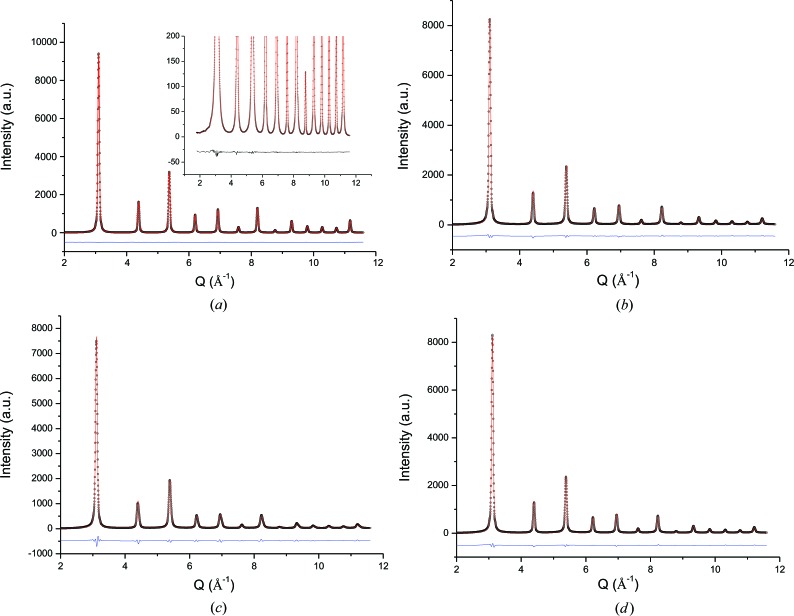
XRPD patterns generated by the Debye scattering equation using atomic coordinates of MD simulations (red circles) with the corresponding WPPM result (blue lines) and their difference (residual, blue lines below each plot) for (*a*) the geometric (ideal perfect crystal) model of grain number 5, with details in the inset, (*b*) the same grain after energy minimization and MD trajectory, (*c*) the same grain as in part (*b*) but with an edge dislocation across it, and (*d*) the same as part (*c*) with a vacancy concentration *c*_v_ = 10^−3^.

To assess the effect of the microstructure on *B*_iso_/MSD, we followed a procedure similar to that of Rebuffi *et al.* (2016 ▸). The XRPD patterns of an ideal powder made up of crystalline domains like those observed in high-resolution TEM images for the ball-milled FeMo powder sample were simulated and investigated. The simulated patterns were treated as ‘experimental’ patterns and analysed by the same WPPM used for the experimental data of the ball-milled FeMo powder. In this case, however, the crystalline domain is known, so the domain size effect can be modelled to perfection using the common volume function (CVF) for the specific polyhedral shape of the grain (Leonardi, Leoni, Siboni & Scardi, 2012 ▸). As an example, Fig. 6 ▸(*a*) shows the ‘experimental’ pattern and WPPM when only the size effect is present, *i.e.* for an isolated (geometric) model of grain number 5, with atomic positions matching a perfect b.c.c. lattice (no MD potential used yet). The match between data and modelling is nearly perfect, showing that the domain size/shape effect is correctly treated by WPPM using the CVF of the studied grain. Fig. 6 ▸(*b*) shows the modelling result after energy minimization and equilibration of the system free of line defects, *e.g.* after 0.6 ns of MD trajectory, a time sufficient to drive the 50-grain cluster into a steady state under the EAM potential at 300 K. The TA powder pattern, obtained from the atomic coordinates of the same grain sampled at several time steps along the MD time trajectory, includes static and dynamic disorder, the former being caused by the grain boundary and grain–grain inter­actions, while the latter is the effect of temperature. Using again the same modelling as for the experimental ball-milled FeMo powder, the refined DW coefficient is *B*_iso_ = 0.4513 Å^2^. This is slightly higher than the experimental value of Fig. 2 ▸(*a*) and the discrepancy can be ascribed both to the model and to the EAM potential, which is not specifically designed and optimized for the thermal properties of iron (Mendelev, 2016 ▸). However, the interesting point is that, when the procedure is repeated on the AP powder pattern, simulated using average atomic coordinates (*i.e.* by averaging coordinates of the same grain sampled at different instants along the MD trajectory) so as to average out all dynamic effects, *B*_iso_ falls to 0.082 Å^2^. This refined value can be taken as an estimate of the static disorder component of *B*/MSD, due to the effect of grain boundary and grain–grain interactions in the 50-grain cluster. *B*_iso_ for the dynamic disorder can be estimated as 0.4513 − 0.082 = 0.369 Å^2^, not far from the experimental value for bulk iron (0.35; Butt *et al.*, 1988 ▸). Therefore, *B*_iso_ from the AP powder pattern is about 20% of *B*_iso_ for dynamic disorder only, in good agreement with the experimentally observed increment of *B*_iso_ and MSRD of ball-milled FeMo with respect to reference bulk Fe.

MD simulations also provide a direct measurement of the MSRD, given by the variance of the distributions centred about each shell in the RPDF of grain number 5.

**Figure 7:**
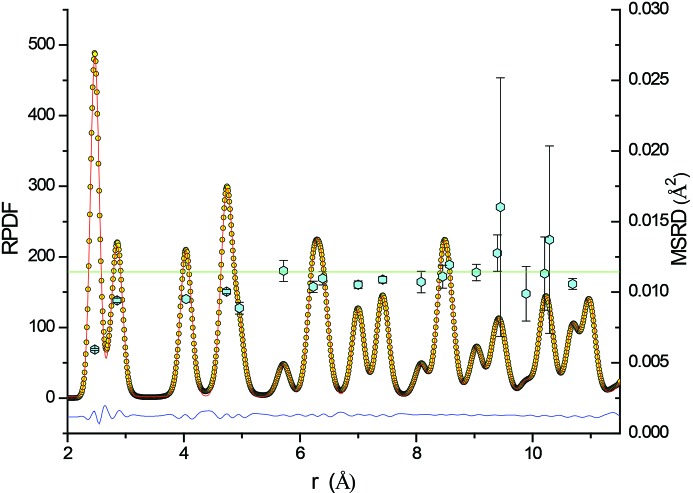
Left axis: the RPDF from MD simulation of grain number 5 (yellow circles) and a fit with Gaussian distributions (red line) centred about the shell radii of neighbouring atoms at increasing distance (first 20 neighbours); the difference (residual) is shown by the blue line below. Right axis: the corresponding MSRD values, given by the variance of the Gaussian distributions (turquoise hexagons), and the asymptotic value, from the refined value of the DW coefficient, *B*_iso_ = 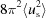 = 0.4513 Å^2^ (green horizontal line).

As shown in Fig. 7 ▸, despite the anharmonicity of the EAM potential, symmetrical Gaussians give an acceptably good fit. As expected, the atomic vibrations of first neighbours are strongly correlated, with the asymptotic value (horizontal line, provided by the refined value of *B*_iso_ = 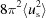 = 0.4513 Å^2^) far from being approached by the MSRD of the nearest-neighbour shells. This trend is in good agreement with that reported by Jeong *et al.* (2003 ▸), with a first-shell DCF about 5% larger than reported by those authors. The discrepancy can be attributed to the different models (MD with EAM potential *versus* the BvK model up to the fifth nearest neighbour) and also to the finite dimensions of grain number 5, as opposed to the perfect bulk Fe of Jeong *et al.* (2003 ▸).

These simulation results add credence to the interpretation that the observed increase in *B*_iso_ is due to grain boundary under-coordination, as also indicated by LEED and Mössbauer experiments (Inagaki *et al.*, 1983 ▸; Clark *et al.*, 1965 ▸; Van Hove *et al.*, 1986 ▸; Von Eynatten & Bömmel, 1977 ▸; Kuwano *et al.*, 1992 ▸) and other studies on plastically deformed mat­erials (Valiev *et al.*, 2000 ▸). The MD approach described above is also useful for assessing the effect of lattice defects of lower dimensionality. Once the grain boundary effect is known, we can repeat the procedure with an edge dislocation created and stabilized by MD inside the same grain (Fig. 5 ▸ *c*). The powder pattern was simulated according to the procedure explained above, to produce ‘experimental’ data now including thermal effects, grain boundary and grain–grain interactions, and the effect of an edge dislocation in the primary slip system of iron. The WPPM result, shown in Fig. 6 ▸(*c*), gives a *B*_iso_ increment of just 2% for the presence of the dislocation.

The same procedure and refinement were then carried out including vacancies in the cluster. With a vacancy formation enthalpy Δ*H*_v_ = 1.41 eV for iron (Kim & Buyers, 1978 ▸), the thermodynamically stable fraction at 300 K would be totally negligible. Even at melting point (1808 K), the vacancy concentration is just *c*_v_ = 1.2 × 10^−4^, which corresponds to only a few vacant sites in grain number 5. In order to give an upper bound to the possible effect of vacancies on *B*_iso_, simulations were done overestimating the concentration, considering extrinsic contributions like the generation of excess vacancies by the dislocation dynamics during plastic deformation. A concentration *c*_v_ = 10^−3^ was used, corresponding to 75 vacant sites over the 66 874 atoms of grain number 5. The result of the modelling of the powder pattern including thermal effects, grain boundary and grain–grain interactions, and the effect of the overestimated vacancy fraction, are shown in Fig. 6 ▸(*d*). Vacancy effects on the modelling were just minor, with no measurable increase in *B*_iso_ (<1%) with respect to the same powder pattern with no vacancies at all.

These results clearly point out the dominant effect of coordination on the value of the DW coefficient. In fact, the experimentally observed increase in *B*_iso_ from reference bulk Fe to ball-milled FeMo matches quite well the static disorder component that MD proved to be caused by a surface effect, where 5642/66 874 atoms are involved in the surface (under-coordinated Fe atoms on the surface of grain number 5). When a full dislocation line is included in grain number 5, that amounts to removing 999/66 874 atoms, with a much lower under-coordination effect than a grain boundary, increasing *B*_iso_ by just 2%. Finally, when randomly removing atoms to create point defects, even well above the equilibrium concentration (75/66 874 atoms), the effects on *B*_iso_ refined by WPPM are below the sensitivity of the technique.

Therefore, there appears to be a hierarchy in the effect of lattice defects on *B*_iso_, mostly determined by the different and increasing number of under-coordinated atoms, following the sequence point–line–surface defects. As shown in this work, and in agreement with earlier research based on surface-sensitive techniques like LEED and Mössbauer, the experimentally observed increase in *B*_iso_/MSD in nanocrystals and plastically deformed nanocrystalline aggregates should not be directly related to a generic ‘microstrain’ effect. The correlation between *B*_iso_ and increased microstrain, frequently proposed in the literature, appears to be just coincidental, as the microstrain frequently increases in inverse proportion to the domain size. The domain size and extension of the grain boundary (or free surface) are indeed the real quantities determining the static disorder for the under-coordination effect of grain boundary or surface atoms.

**Figure 8:**
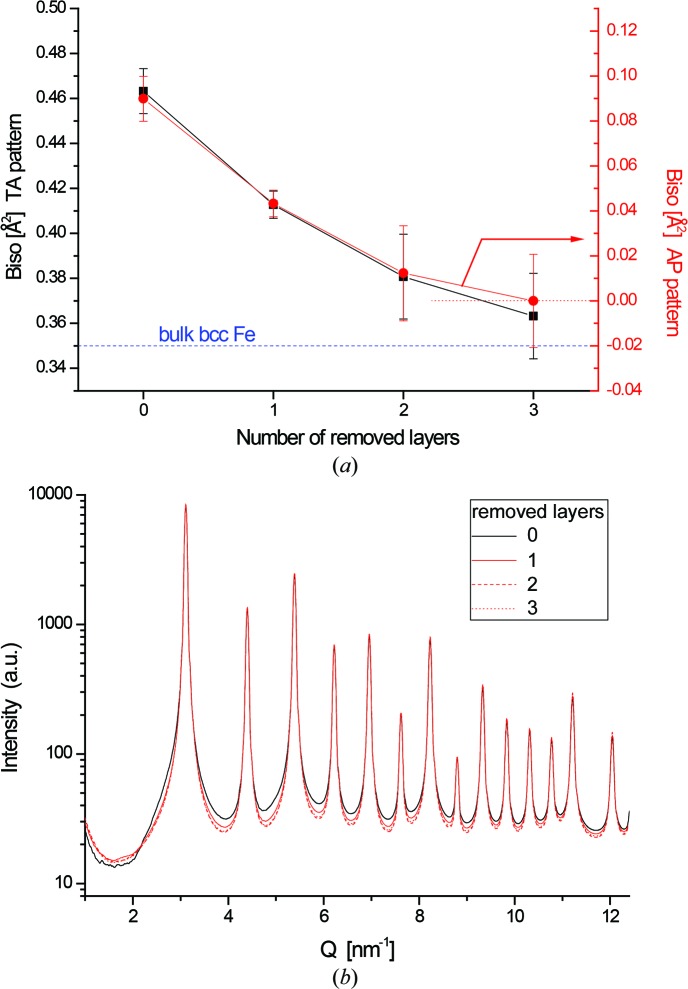
*B*_iso_ values from WPPM of powder patterns generated by the DSE using MD atomic coordinates for grain number 5 after removal of an increasing number of surface layers (from zero to three layers). (*a*) The results are shown for TA patterns (left axis) and AP patterns (right axis), with quite similar trends. The dashed line shows the reference value of *B*_iso_ for bulk iron (Butt *et al.*, 1988 ▸). (*b*) DSE simulated patterns for grain number 5 and after progressive shell removal.

As a further test to assess the contribution of the grain boundary to static disorder and the increased DW coefficient, the powder pattern and WPPM analysis of Fig. 6 ▸(*b*) were repeated after removing one, two or three layers of atoms from the surface of grain number 5. As shown in Fig. 8 ▸, *B*_iso_ decreases quickly with removal of the surface layer, tending to the asymptotic value for bulk Fe. The same decreasing trend is obtained both with TA patterns (which include static and dynamic disorder) and with AP patterns (where the dynamic effect is averaged out). The layer removal procedure has a peculiar effect on the powder pattern. If the patterns are plotted on a log scale to highlight the low-counts region, it is quite evident that removing atomic shells from the grain boundary region inwards drastically reduces the TDS; in addition to a slower decay of the Bragg scattering intensity with *Q*, the patterns show a lower diffuse background when atom layers are removed from the surface. This detail further confirms the importance of a proper evaluation of the TDS if reliable values of the DW coefficient are of interest.

## 5. Concluding remarks   

Synchrotron radiation XRPD and EXAFS patterns from an extensively ball-milled FeMo powder were collected at different temperatures. Measurements and data analysis were carried out paying special attention to all effects influencing the scattered intensity as possible sources of error in the determination of the DW coefficients. The main points of interest are as follows:

(i) Despite the intrinsic differences between the DW co­efficients from XRPD and EXAFS, both show an increase of ∼20% between bulk reference iron and ball-milled FeMo powder; this increment is compatible with experimental observations made by LEED and Mössbauer spectroscopy, which are more sensitive to the surface layers than XRPD and EXAFS and which provide information based on the average over the studied volume of crystalline domains.

(ii) The *B*_iso_/MSD values in our study are markedly lower than those reported in many literature studies based on XRPD; according to the evidence shown by this work, the literature values of *B*_iso_ for nanocrystalline iron appear to be overestimated, either owing to experimental error (*e.g.* because of unaccounted absorption effects) or owing to a lack of consideration of the temperature diffuse scattering.

(iii) The present results, in agreement with the conclusions of LEED and Mössbauer studies, support an interpretation according to which the increased *B*_iso_/MSD in ball-milled nanocrystalline FeMo is mostly caused by the under-coordination of atoms in the grain boundary region. This result is confirmed by atomistic modelling of the studied system. A cluster of plastically deformed nanocrystalline domains of mean size around 8–9 nm was treated by MD using the embedded atom model; the analysis of powder diffraction patterns simulated by the Debye scattering equation using atomic coordinates from MD trajectories demonstrates the main role of surface effects in determining the static disorder contributions to *B*_iso_/MSD.

## 6. Related literature   

For additional literature relating to the supporting information, see Schoonjans *et al.* (2011 ▸) and Caglioti *et al.* (1958 ▸).

## Supplementary Material

Supporting information file. DOI: 10.1107/S160057671700022X/vh5070sup1.pdf
