# Supplementary material for: Debye–Waller coefficient of heavily deformed nanocrystalline iron
Source: J Appl Crystallogr. 2017 Feb 17;50(Pt 2):508–18. doi: 10.1107/S160057671700022X (PMC5377344; doi:10.1107/S160057671700022X)
Supplement: Supplementary file 1 [file j-50-00508-sup1.pdf]

JOURNAL OF
APPLIED
CRYSTALLOGRAPHY

Volume 50 (2017)

Supporting information for article:

Debye-Waller coefficient of heavily deformed nanocrystalline iron

P. Scardi, L. Rebuffi, M. Abdellatief, A. Flor and A. Leonardi

Supporting information

S1. Absorption measurement

The absorption parameter μR (product of linear absorption coefficient and the radius of the capillary) has been determined by measurement with a conventional X-ray source, a vacuum-sealed X-ray tube with Ag anode (emission line $K\alpha_1$ at 22.163 keV), equipped with a Si(111) single-crystal monochromator. The (333) reflection has been used for its narrow bandwidth, in order to properly resolve, by their corresponding scattering angles, the $K\alpha_1$ and $K\alpha_2$ emission lines. At 50 cm from the monochromator a set of vertical and horizontal slits shapes the photon beam in order to reject the $K\alpha_2$ line and, at the same time, limit the beam to 2 (vertically) x 0.1 mm (horizontally) at the sample position, so that the sample (a 300 μm capillary) is able to cover completely the beam.

By putting a photon counter (NaI scintillator) in axis with the photon beam after the sample and moving the sample orthogonally to the beam direction, a stepped scan (step = 0.05 mm) has been performed in order to measure the flux (photons/s) when the absorption from the sample material reach its maximum. The transmittance of the sample has been calculated as the ratio between the flux at maximum absorption and the flux of the beam without the sample (see Figure S1): $T = \frac{F_{\max}}{F_0}$.

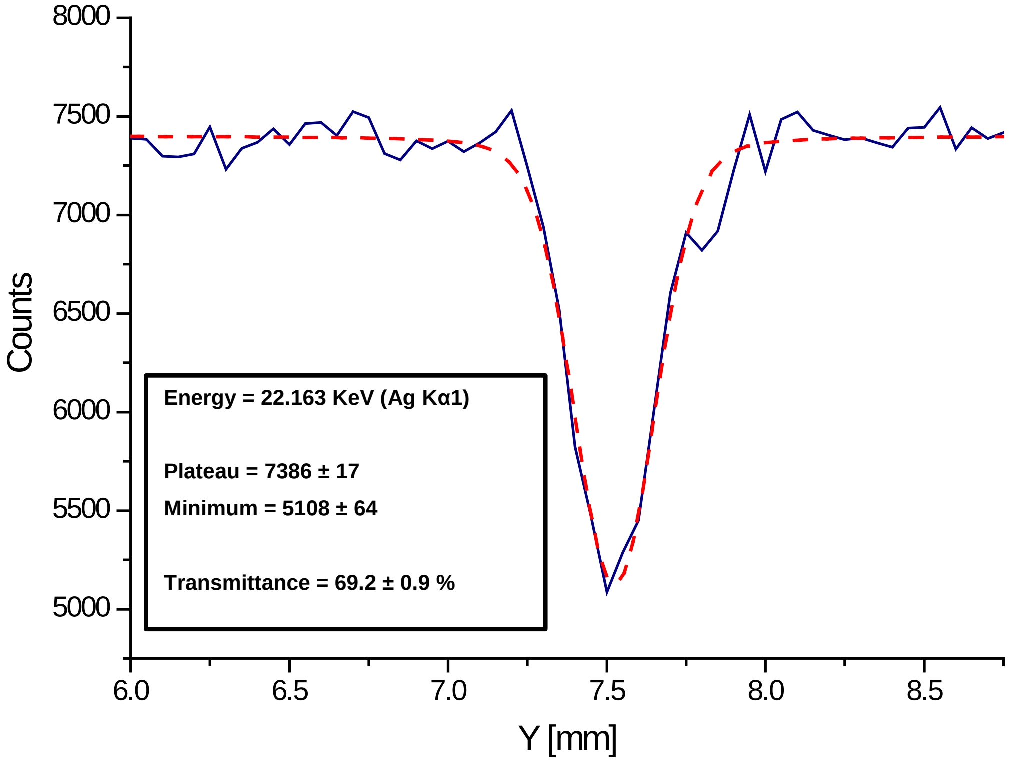

Figure S1 Absorption measurement by stepped horizontal scan (Y, in mm). Minimum is reached when the sample darkens the photon beam completely, and the center of the capillary is positioned at the center of the beam. A Gaussian fit of the detected pattern gives an estimate of the Flux of the beam without sample (F_0), and the Flux at maximum absorption ($F_{\max}$).

The absorption coefficient is calculated by the following equation:

$$\mu(E) = \left[\alpha(E) \cdot \rho\right] \cdot f \qquad \text{(S1)}$$

where $\alpha(E)$ is the mass-energy absorption cross-section in $cm^2 \cdot g$, ρ is the material density in $cm^{-3} \cdot g$, and f is the so-called packing factor, the fraction of volume occupied by the powder inside the capillary (typically ~0.6).

By approximating the central part of the capillary, in the position of maximum absorption, as a parallelepiped with length equal to two times the capillary radius, we can calculate the packing factor with the following relation:

$$T = e^{-2\mu R} = e^{-2\left[\alpha(E)\rho\right] f \cdot R} \qquad \text{(S2)}$$

from which it is straightforward to obtain f as:

$$f = \frac{\ln(T)}{-2\alpha\rho R} \quad \text{(S3)}$$

The mass-energy absorption cross section and the density have been calculated by using the xraylib library (Schoonjans *et al.*, 2011). From the calculation of the packing factor it is possible to determine the absorption coefficient (See Figure S2).

The measurement of the transmittance at AgKa$_1$ energy gives a packing factor of 7.64% for the studied FeMo powder (diluted in Carbon Black), that leads to μR = 0.08 at 30 keV energy. Such a low μR allows us to disregard absorption effects on the XRPD pattern.

```
import xraylib, numpy

radius = 0.015 # cm
transmittance = 0.692

alpha = xraylib.CS_Total_CP("Fe0.985Mo0.015", 22.163) # energy in KeV

rho_1 = xraylib.ElementDensity(26)
rho_2 = xraylib.ElementDensity(42)

rho = rho_1*0.985 + rho_2*0.015

packing_factor = numpy.log(transmittance)/(-2*alpha*rho*radius)

# -------------------------------

alpha_final = xraylib.CS_Total_CP("Fe0.985Mo0.015", 30.0) # energy in KeV
mu_final = alpha_final*rho*packing_factor

transmittance_final = numpy.exp(-2*mu_final*radius)
muR = mu_final*radius

print("P.F.                 : " + "{:4.2f}".format(packing_factor*100) + " %" )
print("Final Transmittance: " + "{:4.2f}".format(transmittance*100) + " %")
print("muR                  : " + "{:4.2f}".format(muR))
```

Figure S2 Python 3 code using the xraylib library to calculate μR at the target energy through the measured transmittance at the AgKa$_1$ energy.

S2. Instrumental profile, capillary pattern, and TDS

The Instrumental Profile was obtained by fitting the powder pattern of NIST SRM660a (LaB_6) (Cline, 2000) standard, collected under conditions comparable with those of the ball-milled FeMo powder. Figure S3 shows the result of the fitting with pseudo-Voigt profile functions. Figure S4 shows the

resulting trends of Full Width at Half Maximum (FWHM), according to the traditional Caglioti function (Caglioti, 1958), and of the Lorentzian profile component (η) as a function of 2θ.

Contribution from the kapton capillary and air scattering was assessed by collecting the pattern from an empty capillary; the blank pattern was then fitted by seven pseudo-Voigt functions, deemed sufficient to reproduce empirically the experimental pattern. The resulting model, shown in Figure S2, was used as background in the modelling of the ball-milled FeMo data, using a refinable scaling factor to adapt it.

Contributions of Temperature Diffuse Scattering to the WPPM analysis of patterns collected at 100K, 200K, and 300K are shown in Figure S6. Details on the theory are given in (Beyerlein *et al.*, 2012).

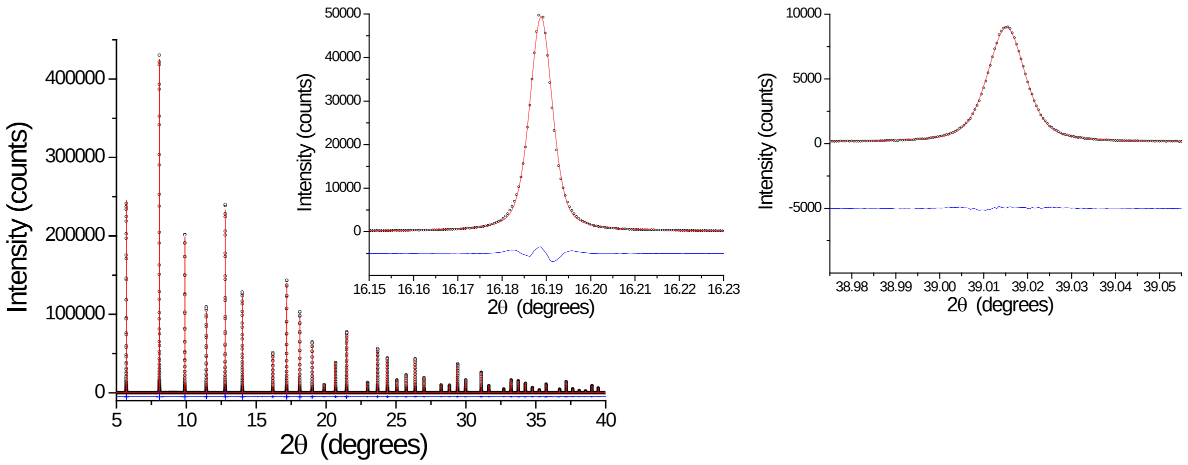

Figure S3 XRPD pattern of NIST SRM660a (LaB_6) (Cline *et al.*, 2000) standard collected at 11bm (Argonne), under conditions equivalent to those used for the FeMo powder of this work. Data (dots) are shown with the result of fitting (line) using pseudo-Voigt curves, with the difference (residual) shown below. Insets show details of instrumental peaks and corresponding modelling.

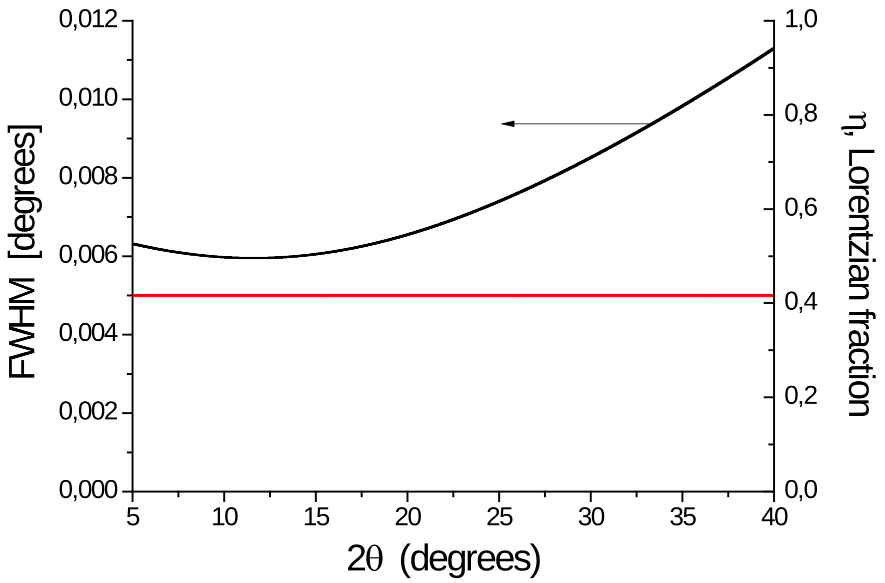

Figure S4 Instrumental profile width and shape from the fitting results of Figure S2. FWHM trend according to the polynomial function of Caglioti *et al.* (Caglioti, 1958):

$FWHM^2 = W + V\tan(\theta) + U\tan^2(\theta)$ (left axis). Lorentzian fraction, η, is practically constant across the angular range considered (right axis).

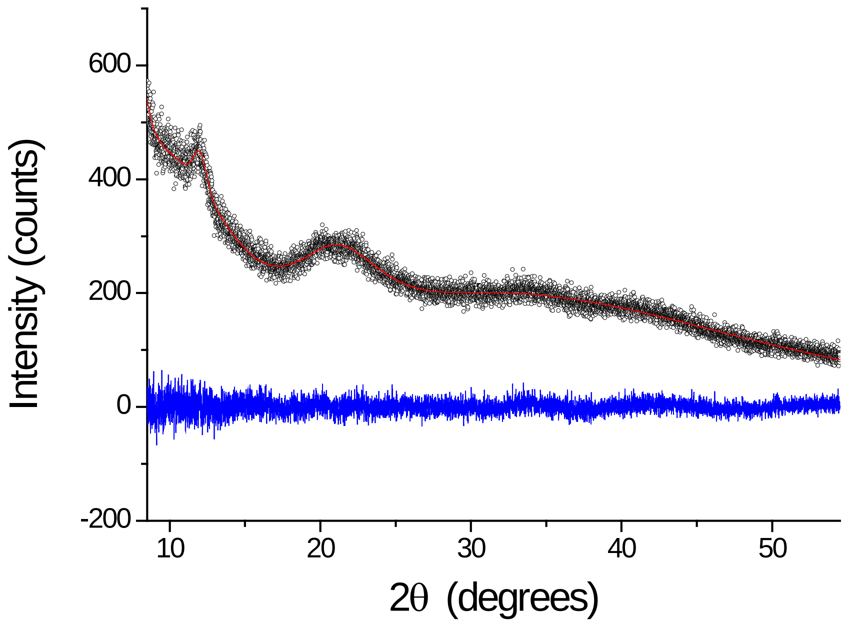

Figure S5 Diffraction pattern of an empty kapton capillary (dot) and fitting with seven pseudo-Voigt functions (line). The difference (residual) is shown below

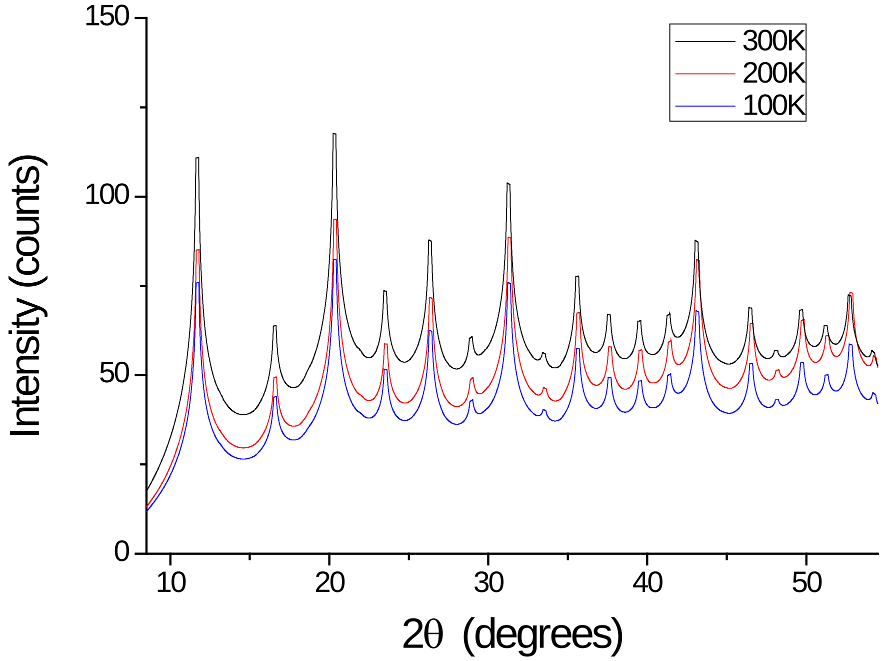

Figure S6 Temperature Diffuse Scattering TDS component for the WPPM analysis of the powder patterns of the ball-milled FeMo samples collected at 100K, 200K, and 300K. Details on the expression used are given in (Beyerlein *et al.*, 2012).

S3. EXAFS

EXAFS signals χ(K) were extracted from the experimental spectra by using a general extraction procedure involving aligning all spectra to a reference sample, and then merging the aligned spectra

for each temperature together to form one averaged spectrum. Then a normalization procedure was done by subtracting linearly the pre-edge and on the other hand using 2nd order polynomial to model the post edge background.

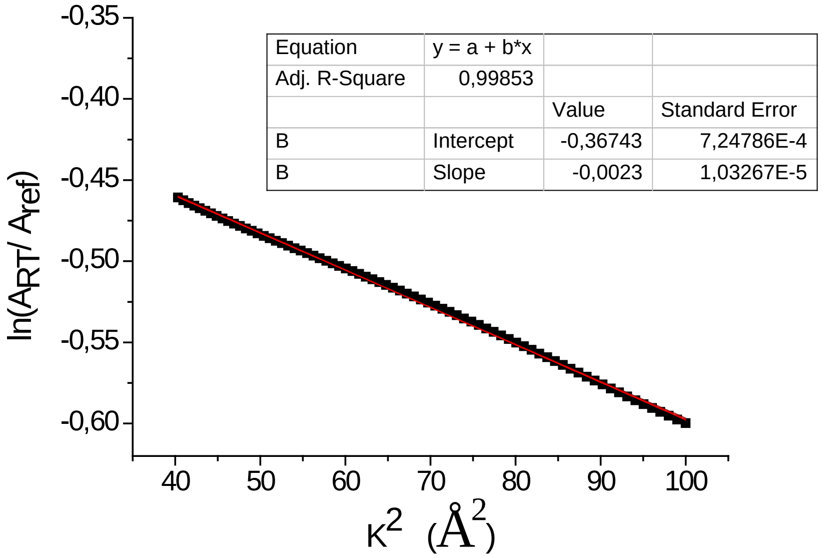

Figure S7 EXAFS data analyzed by the ratio method (Schnohr *et al.*, 2015) at RT (300K)

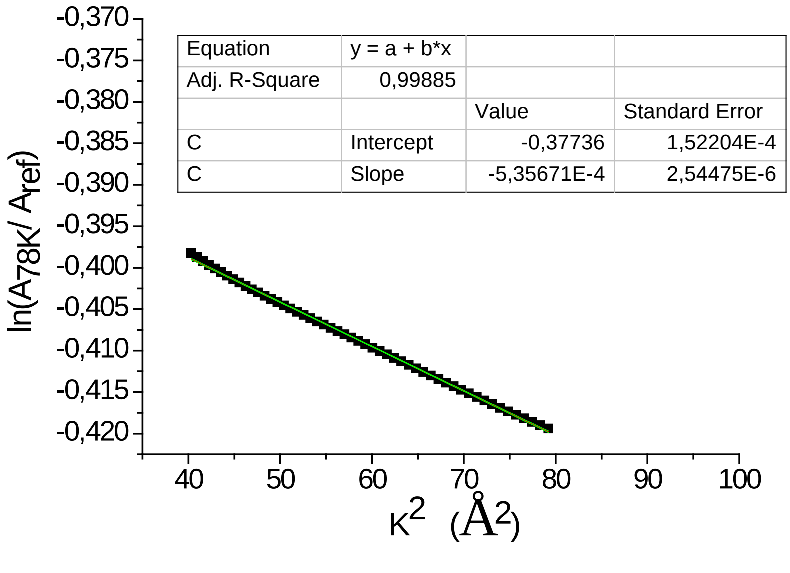

Figure S8 EXAFS data analyzed by the ratio method (Schnohr *et al.*, 2015) at 78K

References:

Schoonjans T., Brunetti A., Golosio B., Sánchez del Río M., Solé V.A., Ferrero C. and Vincze L. (2011). *Spectrochimica Acta Part B: Atomic Spectroscopy* 66, pp. 776-784. doi:10.1016/j.sab.2011.09.011

Caglioti, G., Paoletti, A., Ricci, F.P., (1958). *Nuclear Instruments and Methods* **3**, 223-228

Beyerlein, K. R., Leoni, M. & Scardi, P., (2012). Acta Crystallogr. Sect. A 68, 382–392.

Cline, J. P., Deslattes, R. D., Staudenmann, J.-L., Kessler, E. G., Hudson, L. T., Henins, A. & Cheary, R.W. (2000). Certificates SRM 640c and SRM 660a. NIST, Gaithersburg, MD, USA.

Schnohr, C.S., & Ridgway, M.C., (eds.), (2015) “X-Ray Absorption Spectroscopy of Semiconductors”, Springer-Verlag, Berlin Heidelberg, Ch. 1.
